# Closed-loop control of trunk posture improves locomotion through the regulation of leg proprioceptive feedback after spinal cord injury

**DOI:** 10.1038/s41598-017-18293-y

**Published:** 2018-01-08

**Authors:** Eduardo Martin Moraud, Joachim von Zitzewitz, Jenifer Miehlbradt, Sophie Wurth, Emanuele Formento, Jack DiGiovanna, Marco Capogrosso, Grégoire Courtine, Silvestro Micera

**Affiliations:** 1 Bertarelli Foundation Chair in Translational Neuroengineering, Center for Neuroprosthetics and Institute of Bioengineering, Swiss Federal Institute of Technology (EPFL), CH-1202 Geneva, Switzerland; 2 International Paraplegic Foundation Chair in Spinal Cord Repair, Center for Neuroprosthetics and Brain Mind Institute, Swiss Federal Institute of Technology (EPFL), CH-1202 Geneva, Switzerland; 3 University of Fribourg, CH-1700 Fribourg, Switzerland; 4 The BioRobotics Institute, Scuola Superiore Sant’Anna, 56127 Pisa, Italy

## Abstract

After spinal cord injury (SCI), sensory feedback circuits critically contribute to leg motor execution. Compelled by the importance to engage these circuits during gait rehabilitation, assistive robotics and training protocols have primarily focused on guiding leg movements to reinforce sensory feedback. Despite the importance of trunk postural dynamics on gait and balance, trunk assistance has comparatively received little attention. Typically, trunk movements are either constrained within bodyweight support systems, or manually adjusted by therapists. Here, we show that real-time control of trunk posture re-established dynamic balance amongst bilateral proprioceptive feedback circuits, and thereby restored left-right symmetry, loading and stepping consistency in rats with severe SCI. We developed a robotic system that adjusts mediolateral trunk posture during locomotion. This system uncovered robust relationships between trunk orientation and the modulation of bilateral leg kinematics and muscle activity. Computer simulations suggested that these modulations emerged from corrections in the balance between flexor- and extensor-related proprioceptive feedback. We leveraged this knowledge to engineer control policies that regulate trunk orientation and postural sway in real-time. This dynamical postural interface immediately improved stepping quality in all rats regardless of broad differences in deficits. These results emphasize the importance of trunk regulation to optimize performance during rehabilitation.

## Introduction

Leg sensory feedback circuits play an important role in the generation and regulation of leg movements^1–3^. In healthy conditions, descending supraspinal commands continuously tune the dynamics of these circuits to ensure that movement-related afferent inputs adequately adjust gait patterns^4–6^. After spinal cord injury (SCI), the descending sources of modulation are severely disrupted. Consequently, sensory feedback signals become the primary source of control to produce and regulate leg movements after SCI^7–10^.

The objective of gait rehabilitation therapies is to steer the functional reorganization of spared sensory pathways and residual descending projections through task-specific physical training in order to improve recovery^11–14^. Repeated activation of sensory feedback circuits during standing and walking promotes activity-dependent reorganization of neural connections that ameliorates locomotor performance^7,15,16^. In particular, various studies showed that proprioceptive feedback circuits play a pivotal role in guiding motor execution and circuit reorganization after SCI. For example, epidural electrical stimulation of the lumbar spinal cord specifically modulates proprioceptive feedback circuits associated with extension and flexion of the legs, which enabled refined control of leg motor patterns during gait^17–19^. Moreover, we found that mice lacking muscle spindle feedback circuits fail to display the activity-dependent reorganization of neural pathways that support recovery after SCI^20^. These findings stress the importance of targeting proprioceptive feedback circuits in the design of rehabilitative strategies.

The critical role of movement-related sensory information to steer recovery has motivated the design of training protocols, robotic interfaces and neuroprosthetic systems that predominantly focus on reinforcing reproducible leg movements during rehabilitation^21–24^. In these scenarios, the trunk is typically constrained within bodyweight support systems providing vertically restricted forces, or exoskeletons that constrain pelvis movements. However, natural locomotion involves precisely-timed trunk movements in multiple directions^25^, which directly determine leg biomechanics, and consequently leg sensory feedback during locomotion^26–29^. Indeed, therapists commonly seek to adjust pelvis movements manually during rehabilitation. When possible, they also provide cues to the trunk in order to reinforce the interplay between trunk posture and leg biomechanics. However, the lack of technologies to assist these movements limits the spectrum of possibilities offered to therapists during rehabilitation. The development of rehabilitation protocols and robotic systems that actively regulate trunk posture during training relies on a deeper understanding of the interactions between trunk posture, proprioceptive feedback circuit modulations and leg motor pattern production during locomotion.

Here, we aimed to address these combined aspects in a well-controlled SCI rodent model of bipedal locomotion. First, we designed and fabricated a robotic postural interface that allows real-time control of mediolateral trunk orientation during locomotion in rats with severe spinal cord injury. This robotic system uncovered robust relationships between mediolateral trunk orientation and the bilateral modulation of leg motor patterns during locomotion. We next used a neurobiomechanical computational model of muscle spindle feedback circuits to study some of the mechanisms underlying these modulations. We found that mediolateral trunk orientation modulates the flow of information in muscle spindle feedback circuits. In turn, optimal locomotor performance emerged when mediolateral trunk orientation helped preserve the balance between muscle spindle feedback circuits associated with extensor and flexor muscles for both limbs. This knowledge guided the design of control algorithms that regulated mediolateral trunk orientation in real-time based on subject-specific deficits. Compared to static trunk orientation, this targeted strategy improved locomotor performance across a broad spectrum of gait asymmetries and motor deficits. These results provide an important proof of concept that stresses the need to develop similar dynamic trunk assistance during gait rehabilitation in humans.

## Results

### A closed-loop robotic interface for controlling trunk posture

**Figure 1:**
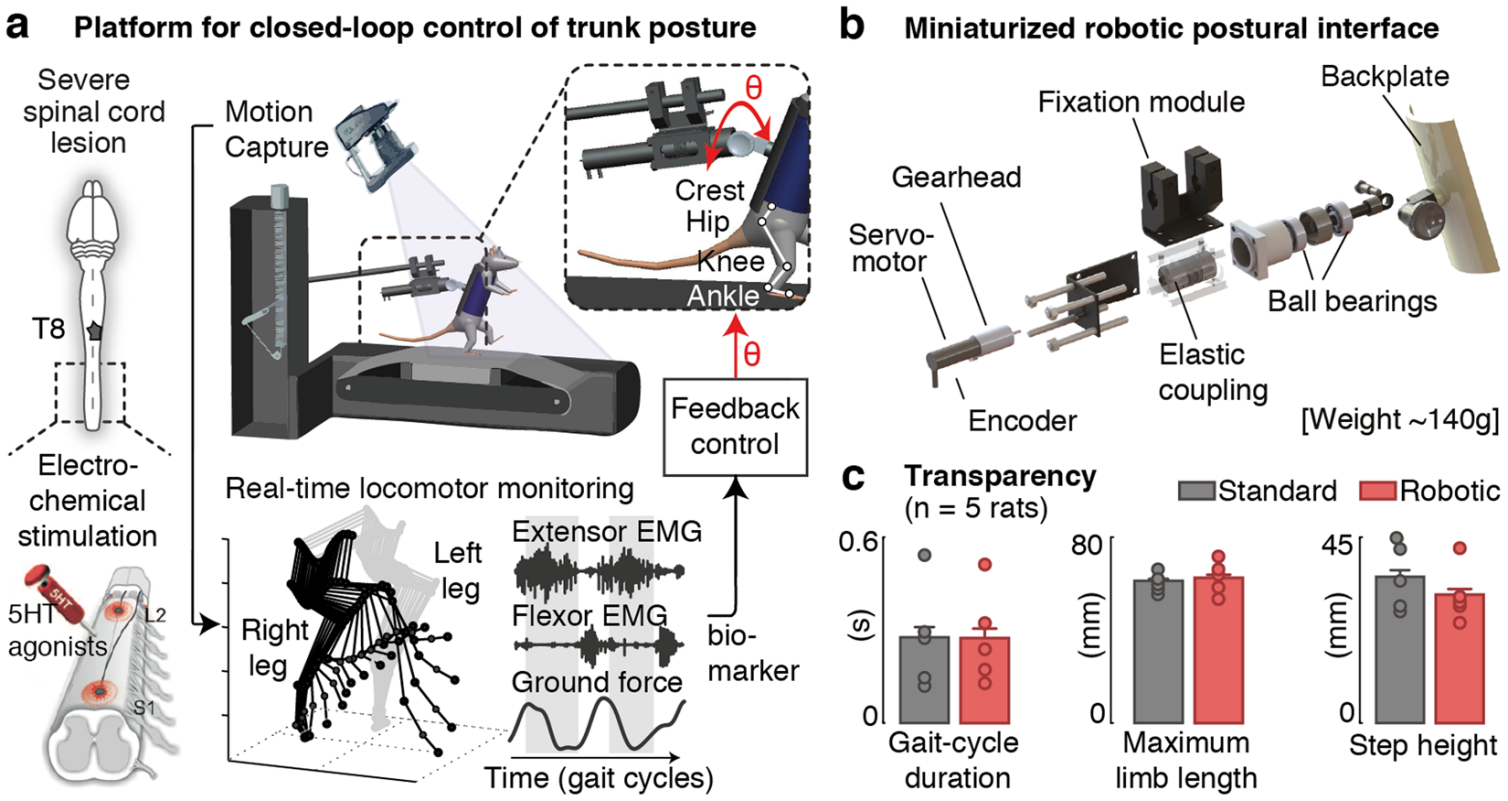
Closed-loop robotic interface for online posture control. (**a**) Integration of the robotic interface within a real-time monitoring platform for closed-loop postural control in paralyzed rats. Animals received a severe contusion of the spinal cord, and were trained to stand and step on a treadmill with body-weight support under electrical and pharmacological spinal cord neuromodulation. Bilateral kinematics, extensor (Medial Gastrocnemius) and flexor (Tibialis Anterior) electromyographic signals and ground reaction forces are recorded synchronously and available online (200 Hz) during continuous stepping. (**b**) Attachment system and embedded components to provide mediolateral actuation with minimal inertial effects. (**c**) Bar plots comparing various locomotor features using the robotic vs. standard non-actuated attachment systems. Data are mean + SEM.

We developed a closed-loop robotic postural interface that supports the control of trunk orientation and postural sway in the mediolateral direction during bipedal locomotion in rats (Fig. 1). To enable the actuation of trunk orientation with minimal inertial effects, we developed a system whose weight remained within the range of existing non-actuated attachment systems (<150 g). We designed a minimal lightweight frame structure and selected miniaturized components to provide sufficient torque in the coronal plane (47.8 mN·m) (Fig. 1), and we integrated this postural interface within a commercially available bodyweight support system (Robomedica Inc, USA). The attachment of the rats to the backplate and the control of the vertical support remained unchanged. We then incorporated the robotic postural interface onto a real-time platform that allows online control of trunk orientation based on bilateral leg kinematics, muscle activity and ground reaction forces (Fig. 1).

To validate the transparency of the system during locomotion, we recorded gait patterns in rats when attached to the robotic postural interface and compared them to standard non-actuated conditions. Rats (n = 5) received a severe lesion of the spinal cord contusion that led to leg paralysis. To enable locomotion, we delivered an electrochemical neuromodulation therapy to the lumbar spinal cord according to methods described previously^15^ (Fig. 1b). Detailed analyses of gait kinematics and kinetics did not reveal significant differences between both support conditions (p > 0.05 across conditions for each rat, Ranksum test, Fig. 1c).

### Adjustment of trunk posture corrects locomotor asymmetries after SCI

We then used this robotic postural interface to characterize the impact of trunk orientation on gait patterns. We mapped the relationships between trunk orientations (angles from −20 to +20 degrees, steps of +/−5 degrees) and the resulting changes in bilateral leg kinematics, muscle activity and ground reaction forces.

Prior to stepping, we calibrated the attachment of each rat to the backplate ensuring that both feet touched the treadmill belt symmetrically. This posture was defined as the baseline trunk orientation.

**Figure 2:**
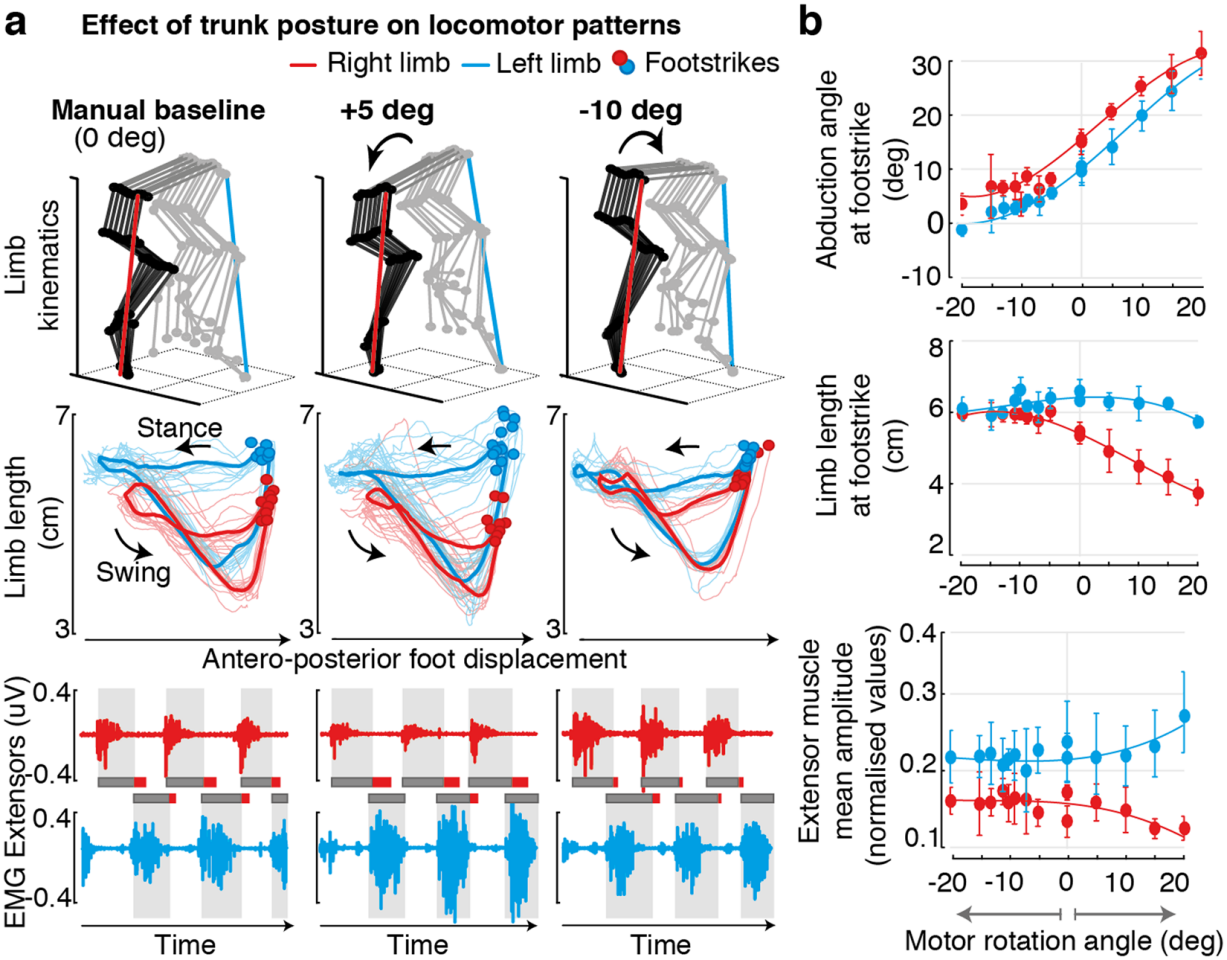
Characterization of postural-effects on locomotor patterns. **(a)** Representative stick diagram decomposition of hindlimb movements, bilateral limb length profiles and extensor EMG traces for one rat under different postural rotations. ‘Baseline’ corresponds to the posture manually defined by therapists during calibration prior to stepping. The animal exhibited weakness on his right limb and asymmetric stepping as a result of the contusion lesion. **(b)** Comprehensive mapping of changes in locomotor features for trunk rotations ranging from −20 to 20 degrees for the same animal (n > 10 consecutive gait cycles for each condition). Data are means + SD.

During stepping, rats exhibited variable gait asymmetries that emerged from well-known differences in the performance of the left and right legs after severe spinal cord injury. For example, Fig. 2 shows the gait pattern from a rat whose right leg was weaker than the left. Due to this inter-limb asymmetry, the weak leg failed to provide enough support during stance and remained continuously over-flexed, while the stronger leg exhibited an over-extended configuration (Fig. 2a). Changes in trunk posture induced gradual modulations in gait patterns, inversely proportional for the right and left legs, which compensated for these deficits. Concretely, rotations towards the stronger leg reinforced the loading on this leg during stance. Concomitantly, it reduced the load on the weak leg, which facilitated foot unloading and improved limb excursions during swing (Fig. 2a,b). In doing so, trunk orientation naturally corrected the abduction angles for both legs. To capture this modulation, we extracted the angle of the whole limb (line connecting the hip to the foot) with respect to the direction of gravity in the mediolateral direction (Fig. 2a). We found robust, monotonic relationships between trunk orientation and this angle for both limbs (Fig. 2b). Together, these adjustments enhanced stepping consistency and gait symmetry (Fig. 2b). In contrast, trunk rotations in the opposite direction exacerbated differences between the right and left legs, which increased gait deficits.

**Figure 3:**
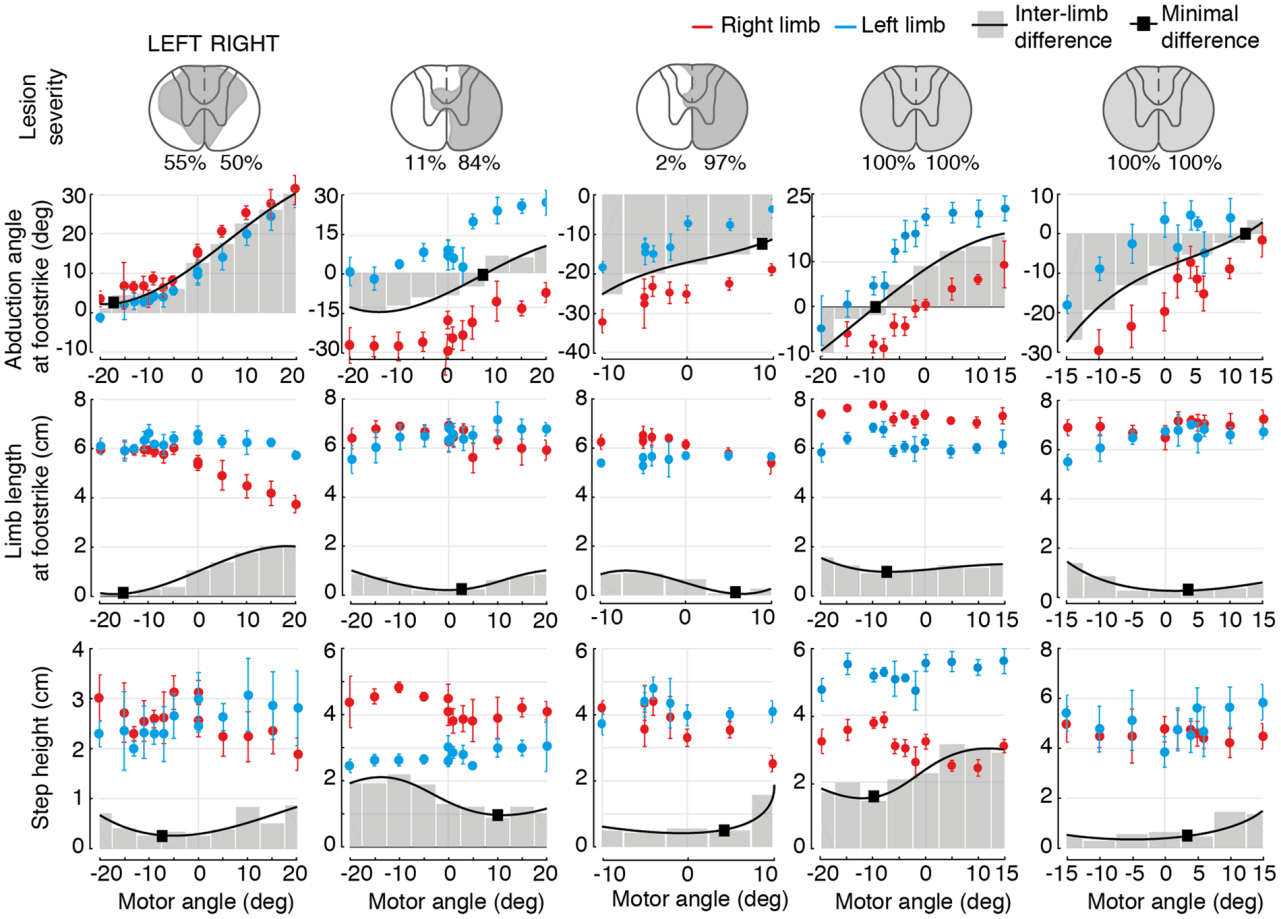
Trunk postural adjustments modulate stepping patterns across animals with different lesion severities. Reconstruction of damaged tissue (shaded areas, including percent) on each hemicord for all animals, and mapping of changes in bilateral locomotor features for each rat for their entire input space. The black line represents the interpolated difference between the right and left limbs. Black squares indicate the minimal difference values across the input space.

Comparisons across rats (n = 5) with different lesion severities confirmed these results (Fig. 3). Regardless of idiosyncratic gait deficits and varying levels of residual supra-spinal control, adjustments of trunk orientation promoted gradual, monotonic changes in whole-limb abduction angles for both legs. These adjustments decreased or increased differences in the limb length and step-height of the left and right legs. For each rat, the more symmetrical gait pattern emerged at a specific degree of trunk orientation in the mediolateral direction (black squares, Fig. 3).

### Trunk posture modulates bilateral muscle spindle afferent dynamics during gait

We next sought to study the neural mechanisms that were likely to underlie the corrections induced by trunk posture on gait patterns. Due to the extensive loss of supra-spinal control, sensory afferent feedback signals were the primary source of control and modulation of leg movements. In particular, muscle spindle afferent feedback circuits are known to play a critical role in the production of locomotion after SCI. These afferents directly encode the changes in limb abduction during trunk rotations. We thus investigated the impact of mediolateral trunk orientation on muscle spindle afferents firing during gait.

**Figure 4:**
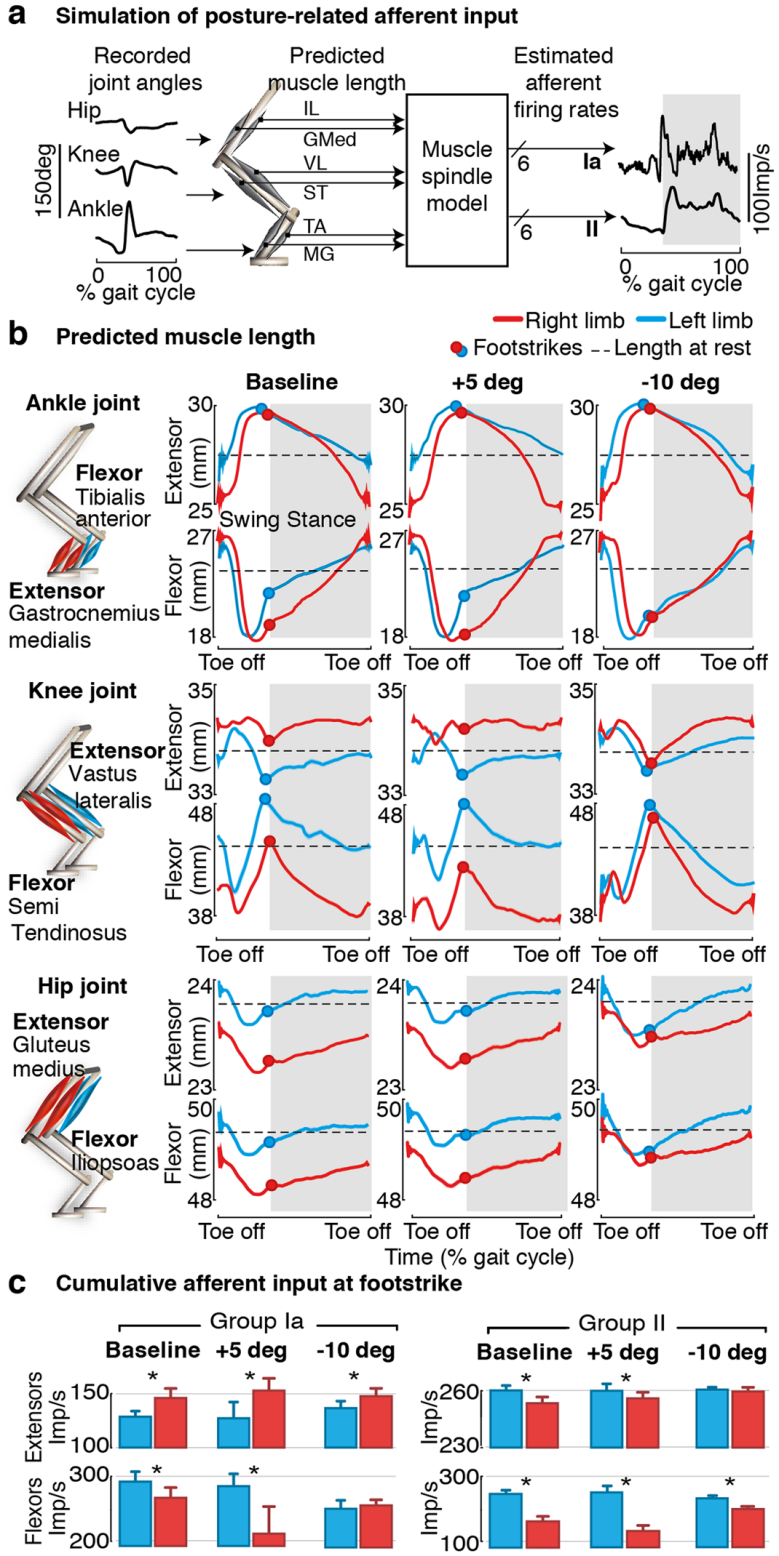
Impact of trunk posture on afferent feedback firing rates. **(a)** Computer simulations of muscle spindle feedback circuit dynamics. Joint angles recorded experimentally are fed into a musculoskeletal model of the rat hindlimb to predict the muscle stretch and stretch velocity profiles for 3 pairs of antagonist muscles (one per joint, for the hip, knee and ankle). Estimated firing rates for group Ia and group II afferent fibers are derived using a muscle spindle model. **(b)** Comparison of estimated right and left stretch profiles throughout the gait-cycle for 3 postural rotations (same as shown in Fig. 2). The red and cyan dots indicate the time of foot-strike events for each limb. **(c)** Bar plots reporting the cumulative flexor- and extensor-related afferent firing rates at foot-strike for each trunk posture. Data are means + SD. *p < 0.05 two-tailed T-test (n = 11 gait cycles).

For this purpose, we employed a dynamic computational model of muscle spindle feedback circuits^18^ to estimate the changes in afferent firing rates for multiple leg muscles. For each trunk orientation, we fed the recorded joint angle trajectories into a realistic 3D biomechanical model of the hindlimb^30^ and we estimated through inverse kinematic the corresponding muscle stretch and stretch velocity profiles for three pairs of antagonistic muscles acting at each joint of the leg (ankle, knee and hip joints) (Fig. 4a). We then used this information to derive the time profiles of group Ia and group II afferent firing rates during gait using a muscle spindle model^31,32^.

Computer simulations revealed that the trunk orientation profoundly altered the stretch profiles of all the simulated leg muscles, in particular those acting at proximal joints (Fig. 4b). At the baseline trunk orientation, inter-limb asymmetries during stepping resulted in marked differences between the stretch profiles of the right and left leg muscles. For example, the profile of ankle flexor and knee extensor muscles diverged strongly at footstrike and during early stance (i.e. for the weak leg, the Tibialis Anterior was under-stretched, while the Vastus Lateralis was over-stretched. The inverse occurred on the strong leg). These differences between left and right stretch profiles led to an excess of extensor-related muscle spindle feedback for the weak leg during stance, and an excess of flexor-related muscle spindle feedback for the strong leg (p < 0.05 between right and left, two-tail T-test, Fig. 4c). Graded adjustments in trunk orientation normalized the stretch profiles in all muscles, which reestablished the natural balance between flexor- vs. extensor-related muscle spindle feedback required to sustain appropriate stepping patterns (Fig. 4b,c).

### Closed-loop control of trunk posture reproduces therapist assistance

The impact of trunk orientation on muscle spindle feedback circuits stressed the importance to optimally define trunk orientation, accounting for deficits that arise throughout movement execution. Commonly, optimal trunk orientations are set empirically by therapists at the beginning of each session, and then remain constant throughout training. We sought to regulate trunk posture automatically based on continuous monitoring of locomotor performance.

**Figure 5:**
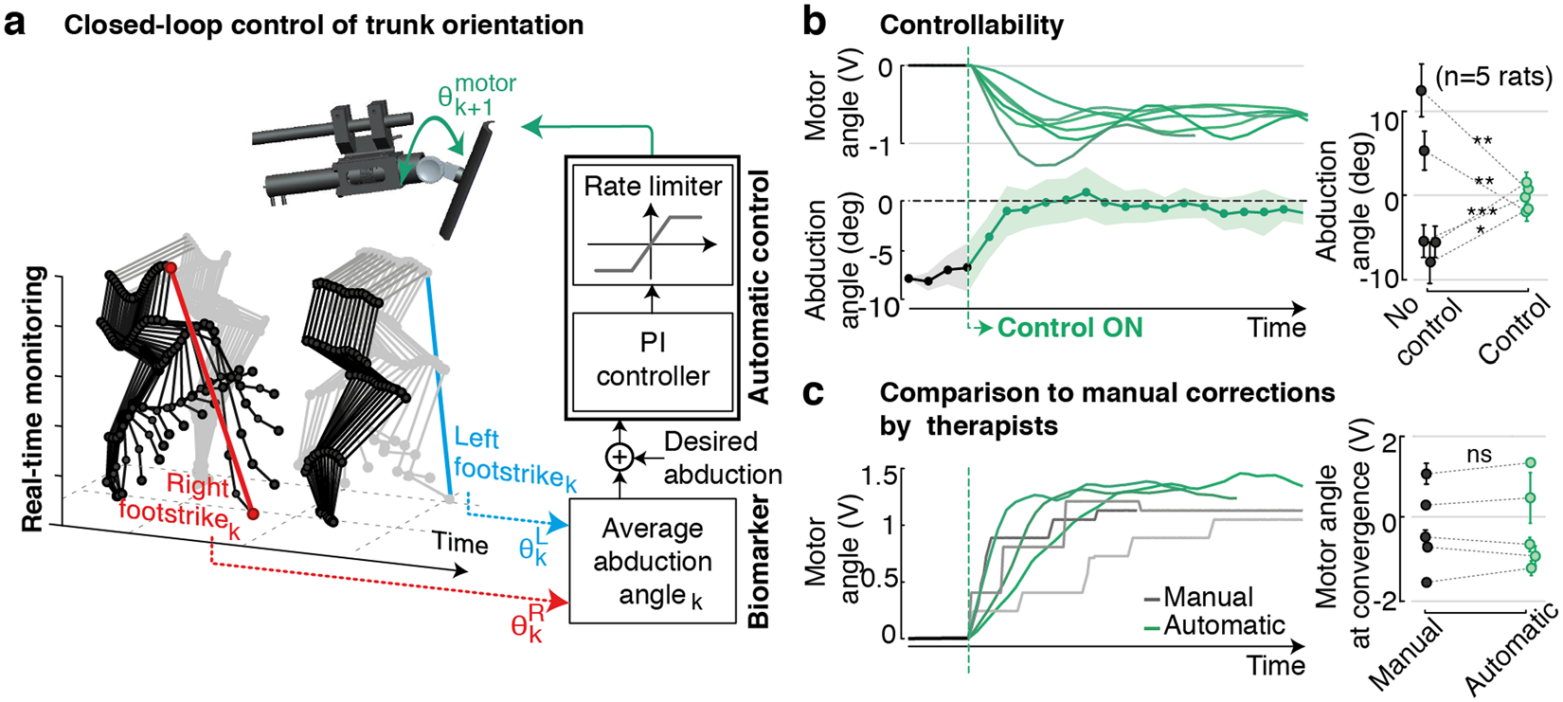
Real-time control of trunk posture during locomotion. **(a)** The closed-loop system continuously monitors bilateral limb kinematics and extracts, for each gait cycle *k*, the mean limb abduction angle at foot-strike (Θ^R^_k_ for the right limb, and Θ^L^_k_ for the left). This biomarker is fed into a PI controller that computes appropriate corrections in trunk rotation Θ^motor^_k+1_ to reduce the error with respect to a desired reference value. **(b)** Performance of the control structure to correct animal-specific deficits. Representative traces of motor rotations and resulting corrections in mean limb abduction for one rat. The desired reference value is set to 0. Bar plots report the comparison of mean abduction angles with and without controlled postural adjustments for all animals (n = 5 rats). **(c)** Example traces comparing automatic and manual trunk corrections provided by experienced therapists for one animal. No significant differences were observed for any rat. Data are means + SD. *P < 0.05, **P < 0.01, ***P < 0.001, ns > 0.05 ranksum test (n > 10 gait cycles).

We previously showed that trunk orientation monotonically modulates the abduction angles of the right and left legs, which in turn correlate with locomotor symmetry (Fig. 3). We thus employed the average of these angles as a biomarker to capture the impact of trunk posture on bilateral stepping performance. We then embedded a feedback control loop within the real-time monitoring platform. A proportional-integer (PI) controller extracted the mean abduction angles of the left and right legs at every gait cycle, and computed the appropriate adjustments in trunk orientation to track a desired reference abduction value (Fig. 5a). Controller parameters were set manually on the first day, and fine-tuned at the beginning of each session based on the behavior of each rat. They remained constant throughout the testing sessions.

To evaluate the performance of this control structure, we quantified its degree of controllability and stability (Fig. 5b). We set the desired reference abduction value to 0 degrees (symmetry) and we evaluated the corrections provided as the controller converged towards the desired value. Regardless of rat-specific deficits or initial trunk orientations, the controller consistently minimized the amount of bilateral abduction and compensated for stepping asymmetries within a few gait cycles (p < 0.05 controlled vs. non controlled conditions for each rat, Ranksum test).

We then verified that the corrections of the controller converged towards optimal values for gait rehabilitation. We asked experienced trainers to fine-tune trunk posture for each rat during a training session in order to optimize locomotor performance, and compared the resulting trunk orientation with the rotation computed by the controller (Fig. 5c). Despite its minimal structure, the closed-loop system provided adjustments that closely matched those of therapists, both in amplitude and speed to converge towards an optimal value (p > 0.05 controlled vs. manual for each rat, Ranksum test).

### Real-time control of postural sway enhances locomotor performance

We finally aimed to capitalize on this robotic interface to restore postural sway during stepping. In healthy subjects, locomotion involves cyclic shifts in bodyweight, which are essential to reinforce loading during stance. The resulting afferent signals reinforce muscle recruitment and contribute to increasing lateral stability. These observations suggest that optimal gait patterns would emerge when reinforcing this aspect of gait at each phase of the locomotor movements.

**Figure 6:**
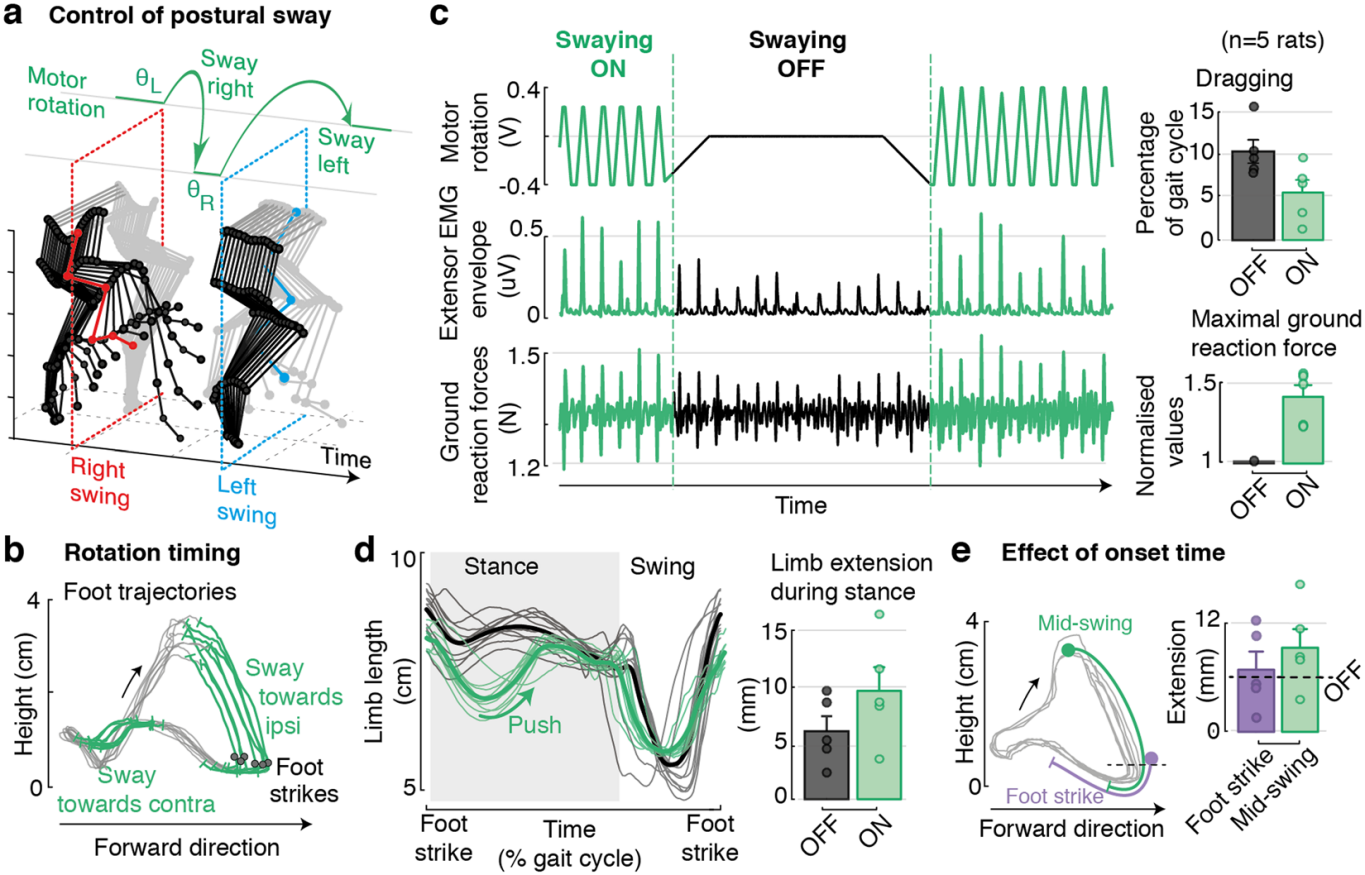
Automatic control of trunk swaying during locomotion. **(a)** An open-loop control logic provides swaying rotations (±5 degrees at constant angular speed) aligned in time with the intrinsic stepping rhythm of each animal. **(b)** Examples of rotation times overlaid on foot trajectories. Adjustments are triggered around mid-swing (concurrently, mid-stance of the contralateral leg) and shift body-weight towards the swinging limb to facilitate swing excursions and reinforce loading at footstrike. **(c)** Representative traces of motor rotations, together with behavioral changes in extensor EMG envelopes and ground reaction forces during a transition between controlled and not controlled phases. Bar plots report changes in the amount of dragging and maximal ground reaction forces for all animals under controlled and non-controlled conditions (n = 5 rats). **(d)** Traces of limb length profiles and quantification of whole-limb extension during the support phase for controlled and non-controlled conditions. **(e)** Comparison of two different swaying onset times (mid-swing and footstrike), and respective increment in limb-length during stance. Data are means + SEM.

To test this hypothesis, we implemented an open-loop control logic that accommodated alternating changes in trunk orientation into the intrinsic stepping rhythm of each rat (Fig. 6a,b). Swaying rotations were centered around the optimal trunk orientation that was previously determined using the proportional-integer controller for each rat. During each stance phase, the robotic interface rotated the trunk by +/−5 degrees at a constant angular speed in the direction of the leg in swing. This postural sway promoted a shift in bodyweight that accompanied the excursion of the swinging limb and reinforced loading at foot contact. Concurrently, these rotations contributed to reducing the weight on the standing leg, and thus facilitated foot unloading at the end of the stance phase.

Compared to static conditions, the delivery of dynamic swaying rotations in trunk orientation immediately increased the amplitude of vertical ground reaction forces and extensor muscle activity (p < 0.05 for each rat, Ranksum test, Fig. 6c). Consequently, the amount of whole-limb extension during stance, as captured by the length of the support leg, significantly increased for each rat (p < 0.05 controlled vs. non controlled for each rat). In parallel, swaying rotations reduced the amount of dragging at foot off for all tested rat (Fig. 6c).

We finally sought to evaluate the importance of the timing at which changes in trunk orientation were delivered (Fig. 6e). The more robust influence on the quality of locomotor movements occurred when rotations were applied from mid-swing to early-stance. Comparatively, rotations triggered at foot strike and not contributing to the ballistic phase of swing mediated a weaker reinforcement of loading and leg extension. Similarly, rotations triggered too early during swing disrupted the support phase occurring on the opposite leg.

## Discussion

Decades of research in gait rehabilitation have illustrated the importance of providing appropriate sensory afferent feedback in order to promote neuroplasticity leading to functional recovery after neurological disorders. Albeit known to play a pivotal role in modulating leg biomechanics, and consequently sensory afferent feedback, the regulation of trunk posture is rarely optimized in training protocols. Here, we provide evidence that trunk posture induced robust, predictable effects on bilateral leg locomotor patterns, which directly impacted the dynamics of proprioceptive afferent feedback circuits. We exploited these results to design closed-loop control strategies that regulated the posture and the dynamical sway of the trunk in real-time based on simple biomarkers. These control policies substantially improved locomotor performance in rats with severe SCI. We discuss these findings with an emphasis on the implications for clinical translation aiming at integrating assistive trunk postural control within gait rehabilitation protocols for humans.

Locomotion involves the synergistic activation of multiple leg and trunk muscles to produce and sustain well-balanced motor patterns. While the legs produce the primary propulsive forces, the trunk actively assists in the coordination of inter-limb movements and the maintenance of equilibrium^33,34^. Particularly critical during bipedal locomotion, the trunk behaves as an inverted pendulum that modulates bodyweight dynamics and propulsion^35,36^. These trunk movements define the amplitude and frequency of alternating gait phases, and in turn shape the resulting leg configuration and muscle recruitment^25,37^. After spinal cord injury, the loss of trunk control adds up to impairments of leg motor control and strongly hinders the capacity to generate stable, coordinated gait patterns^12^. Similarly, constraining pelvis movement in healthy individuals affects gait temporal patterns^28^. The interplay between trunk posture and leg biomechanics^29,36^ underscores the importance of assisting trunk posture during gait training. Our results reinforce this view with quantified outcomes in clinically relevant settings.

During gait rehabilitation, therapists are often forced to focus primarily on leg movements. They seek to correct asymmetries, reinforce weak muscles and prevent compensatory strategies^14^. When possible, they additionally provide manual corrections to pelvis movements, or deliver cues to the trunk in order to facilitate coordinated movements. However, bodyweight support systems and exoskeletons often restrict trunk posture, which strongly limits therapists’ interventions. Our results showed that the regulation of trunk posture naturally addressed many gait deficits without direct assistance of leg movements. We found that gradual adjustments of trunk orientation mediated consistent and predictable modulations of key gait features related to both extension and flexion components, proportionally for each leg, which naturally restored stepping symmetry, reinforced loading and improved stepping quality. Computer simulations suggested that these postural adjustments were sufficient to reestablish the symmetry in muscle stretch profiles for multiple muscles spanning both legs, and thus to restore the appropriate balance between extensor- and flexor-related proprioceptive afferent feedback signals that modulate spinal circuits during stepping^38,39^. Considering the pivotal role of these pathways to steer recovery after injury and their remarkable conservation across mammals^40,41^, we conclude that real-time control of trunk movements to enable well-balanced modulation of proprioceptive feedback may play a critical role for gait rehabilitation in human patients with SCI.

Unlike common gait assistive devices focus on guiding leg movements, which put the emphasis on repeatability at the expense of unrestricted multi-directional movements, the manipulation of trunk posture kept leg movements unconstrained. Modulations in leg biomechanics emerged as a result of natural postural adjustments governing whole-body dynamics. This ecological approach exploited the intrinsic biomechanical properties of the body in order to improve performance^42^, and preserved the step-to-step variability that contributes to increasing spinal neuroplasticity^43^.

Differences throughout training sessions and across animals also stressed the need to compute and adjust optimal trunk orientations for each subject during movement. Asymmetries and dynamic aspects of gait deficits only emerged during locomotion, and could hardly be predicted from the calibration phase prior to stepping. This implies that therapists ought to manually re-adapt the positioning of the subject throughout the training session based on visual inspection, which is suboptimal to mediate maximal therapeutical effects. Here, we showed that real-time control policies are capable of providing continuous assistance of trunk movements automatically with the same level of accuracy as expert therapists.

To elaborate these control policies, we leveraged the reproducible effects of trunk posture on leg motor patterns. Regardless of subject-specific deficits, modulations in limb abduction for the right and left legs consistently captured the adjustments in trunk orientation that were required to promote optimal gait performance. The simplicity and consistency of these relationships within and across sessions supported the design of algorithms with robust performances. A minimal feedback control loop driven by a single input variable and requiring reduced manual tuning of parameters, was sufficient to provide precise corrections of trunk orientation that closely matched therapists’ assistance. Concurrently, we incorporated oscillatory swaying movements in trunk orientation, automatically aligned in time with the intrinsic rhythm of leg motor patterns. This additional trunk assistance immediately reinforced loading, promoted whole-limb extension and reduced dragging. Similarly in humans, therapists sometimes assist the alternating loading of the legs with rhythmic pelvic assistance. In such scenarios, one therapist is located behind the subject, while two additional therapists assist the movements of each leg.

Electrophysiological and experimental observations demonstrated that proprioceptive afferent feedback circuits, in particular from muscle spindles, play a major role in the generation of locomotor behavior^8^, and in guiding functional recovery after injury^20^. These circuits are known to be recruited by epidural electrical stimulation to facilitate motor control^18,44^. Our computer model thus focused on the effects of trunk posture on these circuits. However, trunk postural adjustments undoubtedly modulate afferent pathways conveying gravity-related information to spinal circuits. Particularly critical in humans, inputs for cutaneous and Golgi tendon organ receptors continuously help stabilizing the dynamics governing bipedal gait^45–48^. The inclusion of sensory feedback circuits related to Golgi tendon organs and skin mechanoreceptors in our computer simulations is thus a prerequisite to obtain complete neurobiomechanical insights into the effects of trunk posture on the sensory feedback circuits contributing to the production of gait.

Similarly, we restricted the design of our robotic interface to one actuated degree-of-freedom along the mediolateral direction. Our objective was to provide a proof of principle on the importance of trunk orientation and dynamic trunk movement assistance to enable leg locomotor movements. However, implementing additional actuation capabilities along orthogonal directions will be a key next step to provide optimal postural assistance in clinical settings^49^. In addition to mediolateral trunk movements, well-balanced human locomotion heavily relies on the lateral translations of the pelvis and transverse rotations of the shoulder girdle. These trunk movements help coordinate upper- and lower-body movements and stabilize gait as the center of mass is shifted from side to side^25^. Wearable assistive devices that employ soft materials may provide the flexibility to enable transparent actuation along multiple dimensions for more natural trunk actuation in humans^50,51^.

All these aspects might be especially critical when interfacing trunk assistance with overground gravity-assisted systems, in which propulsion and balance are more engaged than on a treadmill^52^. Our results provide a conceptual and technical framework to design clinically-relevant assistive devices that tailor trunk posture in real-time in order to introduce precision medicine in gait rehabilitation after neurological disorders.

## Methods

### Closed-loop postural robotic interface

#### Hardware design

We established a closed-loop robotic platform to enable precise, transparent actuation of trunk rotations in the mediolateral plane in rats with spinal cord injury (Fig. 1). The design of the postural system aimed to facilitate its integration within commercially available bodyweight-supported treadmill environments for gait rehabilitation. In such environments, animals are endowed with a custom-made upper-body jacket with a Velcro that allows attaching them to a backplate at the tip of the support lever (Fig. 1a). The orientation of the torso can then be manually adjusted and fixated along the medio-lateral and antero-posterior directions with dedicated metallic lockable ball joints. The overall weight of the system is around 150 g.

To ensure the maximal transparency of our actuated robotic system and to facilitate behavioral comparisons, we imposed that our design remains within this same weight range. First, we simplified the design of the system to the absolute necessary requirements. We restricted the actuated rotations to those in the mediolateral plane in order to focus on the impact on lateral stability and sway, and we carefully chose miniaturized components to adjust the torque and precision to the specificities of experiments in rodents (~250–300 g). Actuation was provided using a servomotor (Faulhaber 1028E012B) in combination with a zero-backlash gearhead (Faulhaber 12/5-161:1) which provided a theoretical output torque of 47.8 mN·m (Fig. 1 and Table 1). To measure the absolute position of the motor, we employed a magnetic encoder (Faulhaber AESM-4096) combined with an elastic coupling to avoid any damage to the gearhead due to torsion, bending or impacts on the rotation axis. Second, we developed a minimal, lightweight frame structure in aluminum that concurrently served as attachment onto the bodyweight-support system and protected the alignment of the different components (Fig. 1a). This frame was designed such that the position of the animal with respect to the treadmill belt was preserved, along with other orthogonal degrees of freedom. All additional components (fixations screws, knob) were realized in aluminum, or were 3D-printed.

**Table 1:** Hardware specifications of the robotic postural interface.

Weight	142 g
Theroretical output torque	47.8 mNm
Nominal voltage	12 V
Maximum output power	8.5 W

#### Real-time software

The control of the robotic postural system was implemented as a custom-made Simulink xPC Target application (The MathWorks, Natick, MA) that communicated with the sensing and actuation components via a Humusoft MF624 I/O board (Humusoft s.r.o., Slovakia). To enable real-time control of posture based on the animal locomotor performance, we interfaced this application with a high-speed motion capture system (Vicon, Oxford, UK) that monitored leg movement in real-time (Fig. 1b).

Reflective markers (1 mm) were attached to bilateral hindlimb landmark joints, i.e. iliac crest, greater trochanter (hip joint), lateral condyle (knee joint), lateral malleolus (ankle) and distal end of the fifth metatarsophalangeal (MTP) joint. Raw 3D positions for each marker were continuously recorded (200 Hz) and imported into the Simulink application through an Ethernet connection (TCP/IP) using a dedicated datastream SDK library (Vicon, Oxford, UK).

Raw signals were filtered using adaptive filters (least mean squares), and processed online to account for missing data resulting from occlusions (interpolation from neighboring markers). Each marker was then assigned a specific label corresponding to the corresponding hindlimb joint. Custom-made rules ensured robust maker labeling along the limb. Transmission latencies through the complete loop remained within 20 ms.

All further control policies were implemented as Simulink blocks within the xPC target application. A dedicated graphical user interface (GUI) allowed to modify control parameters, modes and targets during the experimental session.

#### Feedback control policies and feedforward swaying

We sought to design feedback and feedforward control policies providing automatic adjustments to trunk postural orientation on the basis of online locomotor performance.

To align postural changes to the stepping rhythm of each animal, the real-time system continuously monitored bilateral hindlimb kinematics and extracted key gait events (foot-strikes and foot-off events) (Fig. 5a and Fig. 6a). Foot-strikes were determined for each limb after the vertical displacement of the MTP marker crossed a predefined threshold (15 mm) and the limb length reached a maximum value (corresponding to the beginning of the loading phase). Similarly, foot-off events were detected once the foot exhibited a forward displacement, and crossed a vertical threshold. The detection of these events triggered control computations and subsequent robotic adjustments.

First, we implemented a feedback (proportional integral PI) controller in order to correct asymmetric locomotor patterns and excessive abduction (Fig. 5). At every foot-strike, the monitoring system extracted the mediolateral limb abduction angle for each limb and fed it to the controller, which computed the appropriate corrections based on the average of consecutive right and left angles. Controller parameters Kp and Ki were set manually on the first recording session, and fine-tuned for each animal at the beginning of each session. A rate limiter ensured immediate changes did not exceed a value of 5 degrees.

Second, we developed an open-loop feedforward control logic to enable rhythmic swaying rotations during locomotion (Fig. 6). At pre-defined events throughout the gait-cycle, the real-time system provided controlled rotations at constant speed of +/−5 degrees around the optimal trunk posture for each animal. The control logic was implemented such that right and left rotations alternated, thus ensuring an appropriate behavior even in the case of missed or double detections of gait events.

### Neurobiomechanical computational model

#### Biomechanical model

Estimation of muscle stretch profiles during gait were derived from a realistic musculoskeletal model of the rat hindlimb implemented in OpenSim^53^ and validated experimentally^30,54^. We fed the computer model with crest, hip, knee, ankle, and metatarsophalangeal joint angle traces recorded experimentally in rats under different postural conditions (n > 10 steps) and we calculated the corresponding muscle stretch and stretch velocity profiles through inverse kinematics for 3 pairs of antagonist muscles, i.e., Tibialis Anterior (TA) and Medial Gastrocnemius (GM) for the ankle joint, Vastus Lateralis (VL) and Semi Tendinosus (ST) for the knee joint, and Gluteus Medius (GMed) and Ilipsoas (IL) for the Hip joint (Fig. 4a). Muscles lengths at rest were estimated by feeding the biomechanical model with experimental data from a calibration file in which animals were in the static, resting position prior to stepping.

#### Muscle spindle model

The estimated firing rate profiles of group Ia and group II afferents fibers were computed using a spindle model^31,32,55^. Although this model was originally established from cat electrophysiological recordings, dynamical relationships between muscle stretch and firing rate modulations are expected to be well preserved across mammals. Posture-related effects onto right and left dynamics are in all cases comparable to each other, and thus valid to conclude improvements in symmetry and balance. Firing rates of the different fibers were computed as follows:

1 $$Ia\,firing\,rate=50+2\cdot stretch+4.3\cdot sign(strVelocity)\cdot {|Velocity|}^{0.6}$$

2 $$II\,firing\,rate=110+13.5\cdot stretch$$

To derive the cumulative flexor- and extensor-related afferent input at footstrike for each postural condition **(**Fig. 4c), we defined a window (10 ms) around each footstrike and calculated the mean firing rate (>10 gait cycles in each case) for each individual muscle. We then computed the Normal distribution corresponding to the sum for all three extensor muscles, and similarly for the three flexor muscles.

### Experimental procedures

#### Animal model

All experiments were conducted on adult female Lewis rats (∼200 g, Charles River Laboratories). Rats were housed individually on a 12-hour light-dark cycle, with access to food and water ad libitum. Animals were handled daily for at least two weeks before the surgeries. Animal care was performed twice daily throughout the post-injury period. All procedures and surgeries were approved by the Veterinarian Office of the canton of Vaud, Switzerland. All experiments and methods were performed in accordance with the relevant guidelines and regulations.

#### Surgical procedures and post-surgical care

All surgical procedures have been previously described in detail^7,17^. Interventions were performed under aseptic conditions and general anesthesia (1–3% Isoflurane via facemask) on a heating pad to prevent hypothermia. Three animals received an incomplete lesion (lateral contusion at a mid-thoracic level, ~T8) and three additional animals received a complete spinal cord transection at the same level. One animal of this second group died right after surgery. A partial laminectomy was performed from T7 to T9 in which the spinous processes and the dorsal and lateral aspects of the vertebral column were removed to expose the spinal cord. The dura was opened along the mid-line. Contusions were performed using an Infinite Horizons Impactor (Precision Systems Instruments LCC) with special clamps attached to vertebrae T6 and T10. The spinal cord was impacted by a metal probe (1.3 mm diameter) with a force of 370 ± 30 kDyn. For complete transections, the exposed spinal cord was cut with customized tools. The extent of contusion lesions and the completeness of spinal cord transections were verified histologically post-mortem.

To record electromyographic signals, bipolar electrodes were implanted into extensor (Medial Gastrocnemius) and flexor (Tibilialis Anterior) muscles of the ankle joint. Recording electrodes were fabricated by removing a small part (0.5-mm notch) of insulation from the implanted stainless steel wire (AS631, Cooner Wire). A common ground was created by removing about 1 cm of Teflon from the distal extremity of an additional wire, inserted subcutaneously over the right shoulder. All electrode wires were connected to a percutaneous amphenol connector (Omnetics Connector Corporation) cemented to the skull of the rats. The proper location of the electrodes was verified postmortem.

Stimulating electrodes were created by making a notch (~1 mm) in the insulation of Teflon-coated wires (same as for EMG recordings), which were subsequently secured during surgery at the midline overlying spinal levels L2 and S1 by suturing the wires to the dura. A common ground wire (1 cm of Teflon removed at the distal end) was inserted subcutaneously over the right shoulder. All electrode wires were connected to a percutaneous amphenol connector (Omnetics Connector Corporation) cemented to the skull of the rat.

Analgesia (buprenorphine Temgesic, ESSEX Chemie AG, 0.01–0.05 mg per kg, subcutaneous) and antibiotics (Baytril 2.5%, Bayer Health Care AG, 5–10 mg per kg, subcutaneous) were provided for 5 days post-surgery.

#### Locomotor training and recordings

Starting 7–10 days post-injury, rats were trained for bipedal locomotion for 20–30 minutes every other day. Training took place on a bodyweight-supported treadmill (9 cm·s^−1^, ~40–60% BWS). Hindlimb locomotion was enabled by epidural electrical stimulation applied at S1 and L2 spinal segments (Parameters: 0.2 ms, 40 Hz, 150–300 uA) and systemic administration of pharmacological neuromodulators (Quipazine, 0.2–0.3 mg per kg intra-peritoneal, and 8-OHDPAT 0.05–0.3 mg per kg subcutaneous)^7^. Behavioral recordings started once the animals reached a stable locomotor performance (after ~21 days of training). Characterization and control experiments were performed on subsequent days.

### Statistical Analyses

All data are reported as means ± SEM, unless otherwise specified. Two-tailed T-tests were employed to evaluate differences between normally distributed data (Kolmogorov-Smirnov test), and Ranksum test or two-tailed Kruskal-Wallis test were used for nonparametric evaluations. The statistical a level P < 0.05 was considered significant.

### Data availability

The datasets generated and analyzed during the current study are available from the corresponding author on reasonable request.

## Electronic supplementary material

Supp Video 1
